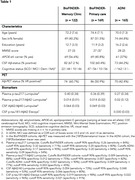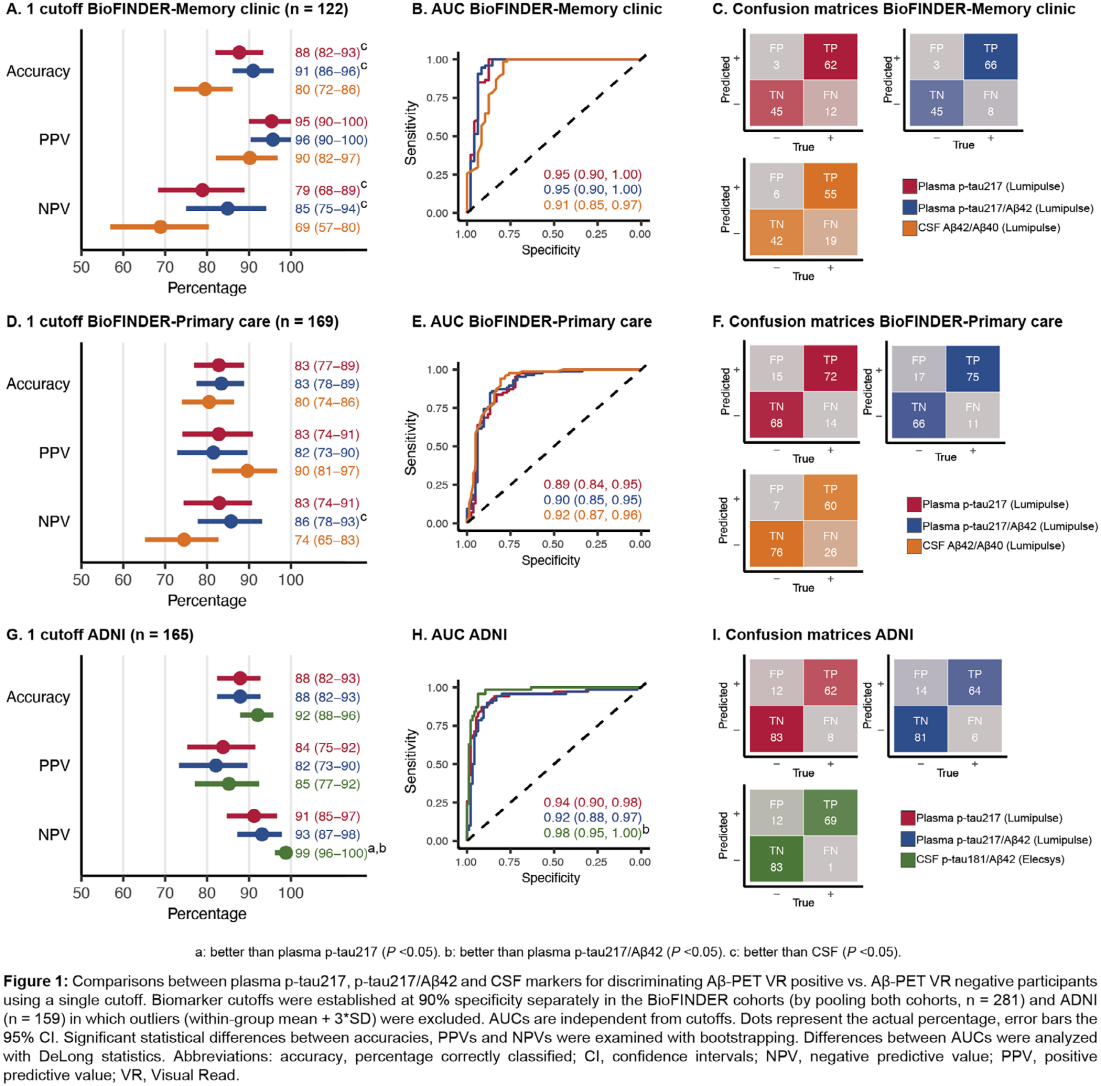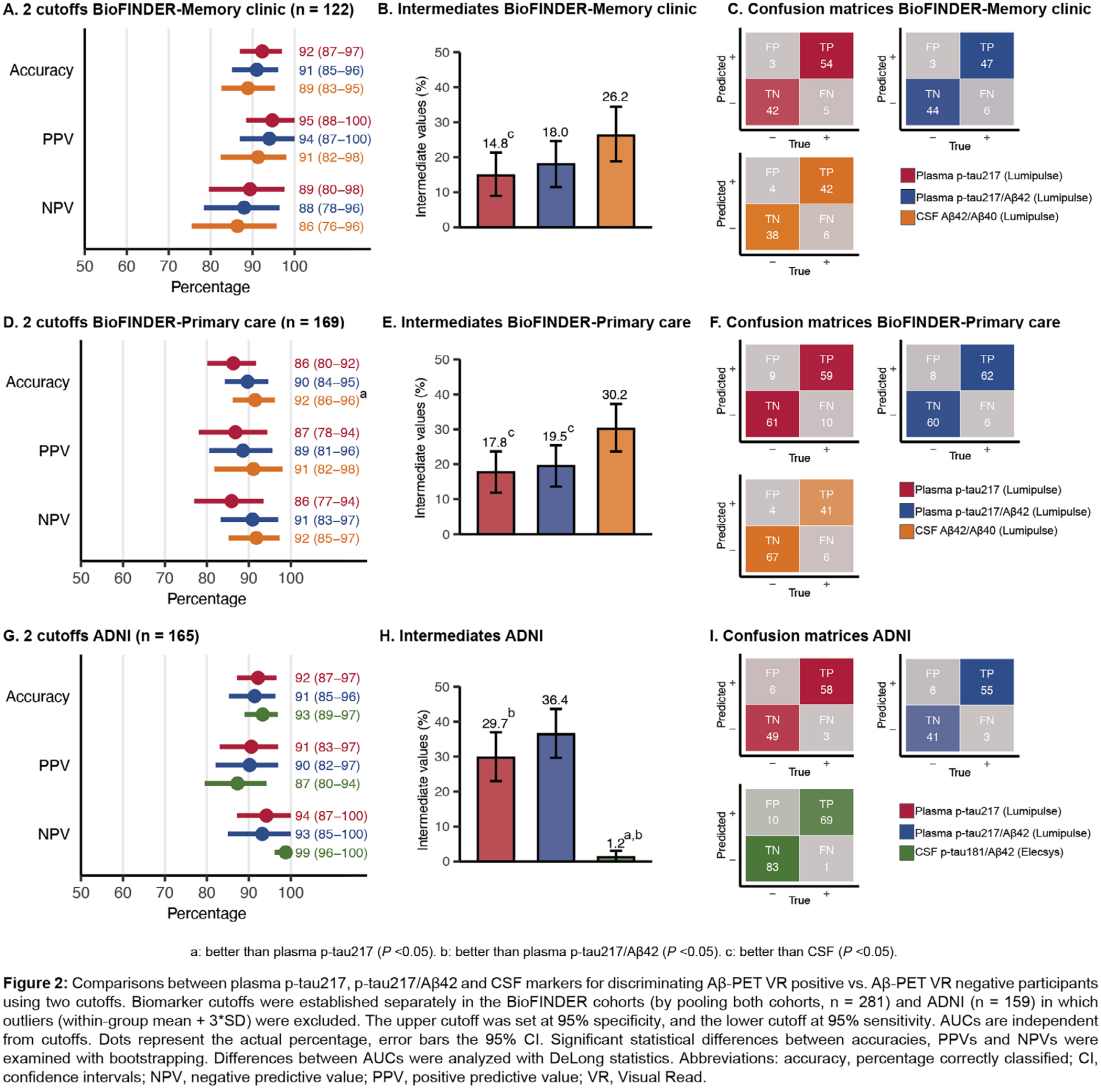# Comparison of plasma biomarkers measured on a fully automated instrument versus CSF biomarkers for detecting Alzheimer’s disease pathology

**DOI:** 10.1002/alz70861_108520

**Published:** 2025-12-23

**Authors:** Noëlle Warmenhoven, Alexa Pichet Binette, Lyduine E. Collij, Shorena Janelidze, Niklas Mattsson‐Carlgren, Rik Ossenkoppele, Pontus Tideman, Alzheimer's Disease Neuroimaging Initiative (ADNI), Nicholas J. Ashton, Henrik Zetterberg, Kaj Blennow, Erik Stomrud, Gemma Salvadó, Oskar Hansson, Sebastian Palmqvist

**Affiliations:** ^1^ Lund University, Lund Sweden; ^2^ Centre de Recherche de l’Institut Universitaire de Gériatrie de Montréal, Montréal, QC Canada; ^3^ Department of Physiology and Pharmacology, Université de Montréal, Montréal, QC Canada; ^4^ Amsterdam University Medical Center (Amsterdam UMC), Amsterdam, North Holland Netherlands; ^5^ Memory Clinic, Skåne University Hospital, Malmö, Skåne Sweden; ^6^ Wallenberg Center for Molecular Medicine, Lund University, Lund Sweden; ^7^ Amsterdam Neuroscience, Brain Imaging, Amsterdam Netherlands; ^8^ Alzheimer Center Amsterdam, Neurology, Vrije Universiteit Amsterdam, Amsterdam UMC location VUmc, Amsterdam Netherlands; ^9^ Laboratory of Neuro Imaging (LONI), University of Southern California, Los Angeles, CA USA; ^10^ Banner Sun Health Research Institute, Sun City, AZ USA; ^11^ King’s College London, Institute of Psychiatry, Psychology & Neuroscience, Maurice Wohl Clinical Neuroscience Institute, London UK; ^12^ Department of Psychiatry and Neurochemistry, Institute of Neuroscience and Physiology, The Sahlgrenska Academy, University of Gothenburg, Mölndal Sweden; ^13^ Banner Alzheimer's Institute, Phoenix, AZ USA; ^14^ Hong Kong Center for Neurodegenerative Diseases, Hong Kong, Science Park China; ^15^ Clinical Neurochemistry Laboratory, Sahlgrenska University Hospital, Mölndal, Västra Götalands län Sweden; ^16^ Wisconsin Alzheimer's Disease Research Center, University of Wisconsin‐Madison, School of Medicine and Public Health, Madison, WI USA; ^17^ Department of Neurodegenerative Disease, UCL Institute of Neurology, London UK; ^18^ UK Dementia Research Institute, University College London, London UK; ^19^ Barcelonaβeta Brain Research Center (BBRC), Pasqual Maragall Foundation, Barcelona Spain

## Abstract

**Background:**

As fully automated instruments for plasma biomarkers for Alzheimer’s disease (AD) are being implemented in specialist clinics, many are questioning whether they can be used interchangeably with, or as substitutes for, gold‐standard cerebrospinal fluid (CSF) biomarkers. We aimed to compare fully automated Lumipulse plasma assays that are under FDA evaluation with FDA‐approved CSF assays.

**Method:**

We included patients with subjective cognitive decline, mild cognitive impairment, and dementia from the BioFINDER‐Memory Clinic (*n* =122), BioFINDER‐Primary Care (*n* =169) and ADNI (*n* =165) cohorts, in which plasma *p* ‐tau217 and plasma Aβ42 were measured with Lumipulse assays (Table 1). Plasma biomarkers were compared with CSF Aβ42/Aβ40 (Lumipulse) in the BioFINDER cohorts and CSF *p* ‐tau181/Aβ42 (Elecsys) in ADNI. Previously published one‐ and two‐cutoff approaches were used for the biomarkers. The two‐cutoff approach applies upper (positivity) and lower (negativity) cutoffs with intermediate cases being those with results in between the cutoffs (excluded in the analyses). The outcome was presence of AD pathology (visual read Aβ‐PET).

**Result:**

Plasma *p* ‐tau217 identified AD pathology with AUCs of 0.95 (memory clinic), 0.89 (primary care) and 0.94 (ADNI). For *p* ‐tau217/Aβ42, AUCs of 0.95 (memory clinic), 0.90 (primary care), and 0.92 (ADNI) were observed (Figure 1B,E,H). CSF performed similar in BioFINDER (AUCs 0.91–0.92; all *p* >0.304) and better in ADNI (AUC 0.98, *p* =0.032). When applying one cutoff, plasma *p* ‐tau217 and *p* ‐tau217/Aβ42 had higher accuracies than CSF Aβ42/Aβ40 in BioFINDER‐Memory Clinic (80‐91%, all *p* <0.024) (Figure 1A), while performances were similar in BioFINDER‐Primary Care (80‐83%, all *p* >0.488, Figure 1D) and ADNI (88‐92%, all *p* >0.152, Figure 1G). With two cutoffs, no significant differences between plasma *p* ‐tau217 (92%) or plasma *p* ‐tau217/Aβ42 (91%) and CSF biomarkers (89‐93%) were observed in BioFINDER‐Memory Clinic or ADNI (Figure 2A,G). In primary care, plasma *p* ‐tau217 (86%), but not *p* ‐tau217/Aβ42 (90%), had lower accuracy than CSF Aβ42/Aβ40 (92%, *p* =0.028; Figure 2E).

**Conclusion:**

Plasma *p* ‐tau217 and *p* ‐tau217/Aβ42 Lumipulse demonstrated similar or slightly inferior performance compared with FDA‐approved CSF tests for detecting AD pathology across three cohorts, including primary care. Since these plasma assays are under FDA‐review for AD diagnosis, the results are essential for validating their performance relative to CSF biomarkers. Additional comparisons will be presented at AAIC.